# A systematic review of telehealth screening, assessment, and diagnosis of autism spectrum disorder

**DOI:** 10.1186/s13034-022-00514-6

**Published:** 2022-10-08

**Authors:** Meimei Liu, Zenghui Ma

**Affiliations:** 1 Vrije University Amsterdam, Amsterdam, The Netherlands; 2 Beijing ALSOABA Technology Co. LTD, ALSOLIFE, Beijing, China

**Keywords:** Telemedicine, ASD, Screening, Diagnosis, Assessment

## Abstract

There is a significant delay between parents having concerns and receiving a formal assessment and Autism Spectrum Disorder (ASD) diagnosis. Telemedicine could be an effective alternative that shortens the waiting time for parents and primary health providers in ASD screening and diagnosis. We conducted a systematic review examining the uses of telemedicine technology for ASD screening, assessment, or diagnostic purposes and to what extent sample characteristics and psychometric properties were reported. This study searched four databases from 2000 to 2022 and obtained 26 studies that met the inclusion criteria. The 17 applications used in these 26 studies were divided into three categories based on their purpose: screening, diagnostic, and assessment. The results described the data extracted, including study characteristics, applied methods, indicators seen, and psychometric properties. Among the 15 applications with psychometric properties reported, the sensitivity ranged from 0.70 to 1, and the specificity ranged from 0.38 to 1. The present study highlights the strengths and weaknesses of current telemedicine approaches and provides a basis for future research. More rigorous empirical studies with larger sample sizes are needed to understand the feasibility, strengths, and limitations of telehealth technologies for screening, assessing, and diagnosing ASD.

## Introduction

Autism spectrum disorder (ASD) is a neurodevelopmental condition characterized by impairments in social communication and interactions and repetitive and restricted patterns in behaviors, interests, and activities [24, 26]. The prevalence of ASD has steadily increased over the last two decades and is now estimated to be approximately 1% globally [17, 18, 24, 25]. Despite the documented high prevalence of ASD and the benefits of early intervention, the average age at diagnosis remains high. For example, the average diagnosis age is over 4 years in the United States (US), and approximately 27% of children with ASD remain undiagnosed at age 8 [9].

Several factors contribute to the delayed diagnosis of ASD. The first factor may be the rapidly growing demand for early screening and diagnosis. This time-intensive process heavily relies on a limited number of pediatric specialists [16]. Furthermore, the rising prevalence of ASD increases the strain on these limited specialists and primary care professionals, resulting in extended wait times [16]. The delayed diagnosis phenomenon can further be affected by the low capability of primary care settings to diagnose ASD. In the US, only 1% of patients with ASD are diagnosed by healthcare providers, probably due to the lack of specialty training and low confidence in diagnostic instruments [38].

Another factor could be that ASD is a highly heterogeneous disorder; thus, diagnosing ASD is particularly challenging. Cooccurring psychiatric or neurological disorders are prevalent in individuals with ASD, potentiating the diagnostic challenge [21, 35]. Unfortunately, the global coronavirus disease (COVID-19) pandemic has further complicated ASD diagnostics. Healthcare services may be disrupted during the pandemic due to insufficient equipment and lockdowns. Moreover, patients may be concerned about the risk associated with the virus to children (especially those with intellectual and developmental disabilities) and older individuals [22, 46]. Therefore, telemedicine is a potential approach for improving the efficiency of early screening and diagnosis of ASD.

Telemedicine is the practice of medicine using technology to deliver care at a distance. Telemedicine enables medical professionals to care for a patient at a different location using a telecommunications infrastructure from a central location. Telemedicine could be an effective alternative to in-person ASD screening, assessment, and diagnosis [31]. Telemedicine has distinct advantages over conventional face-to-face methods for the early detection and diagnosis of ASD. For example, digital medical devices can help address the rapidly growing need for early screening and diagnosis of ASD and improve the administration standardization level of ASD screening, assessment, and diagnosis.

Digital screening, assessment, and diagnostic devices for ASD are currently available in primary care settings. These digital medical devices enable providers to obtain a positive or negative diagnostic reference for children suspected to have ASD based on observations and questionnaire results from short home videos and online behavioral checklists [28, 29]. As a result, there is a significantly shorter wait between parents expressing concerns and receiving developmental assessments for their children. Additionally, telemedicine applications greatly facilitate primary care providers in diagnosing ASD and/or referring complex cases for specialist review. Therefore, an overview of current digital medical devices used for ASD screening and diagnosis and their psychometric properties is necessary to provide guidance and recommendations for pediatric care providers.

To date, only four reviews have discussed the use of telehealth in screening and diagnosing ASD, and very few studies have been reported in the literature. For example, Alfuraydan et al. [3] reviewed the application of telehealth for ASD diagnosis but not for screening. Other reviews have mainly focused on the psychometric properties of telemedicine for ASD diagnostics [23, 42, 43]. For instance, Desideri et al. [11] reviewed the application of telemedicine for the early screening of children with ASD under 6 years of age along with psychometric properties. However, none of the previous literature reviews have comprehensively examined the application and observed behavioral indicators of telemedicine for the screening and diagnosis of ASD. The current study aimed to close this gap by thoroughly reviewing the telemedicine applications currently available for ASD screening and diagnosis. We cover the technology used, the observed metrics and the assessed scenarios; additionally, the sample characteristics and psychometric properties described in the studies are discussed. We aimed to provide ASD screening and diagnosis application options for use in daily practice by relevant healthcare practitioners and researchers to help diagnose various conditions and suggest possible future research directions.

## Method

**Fig. 1:**
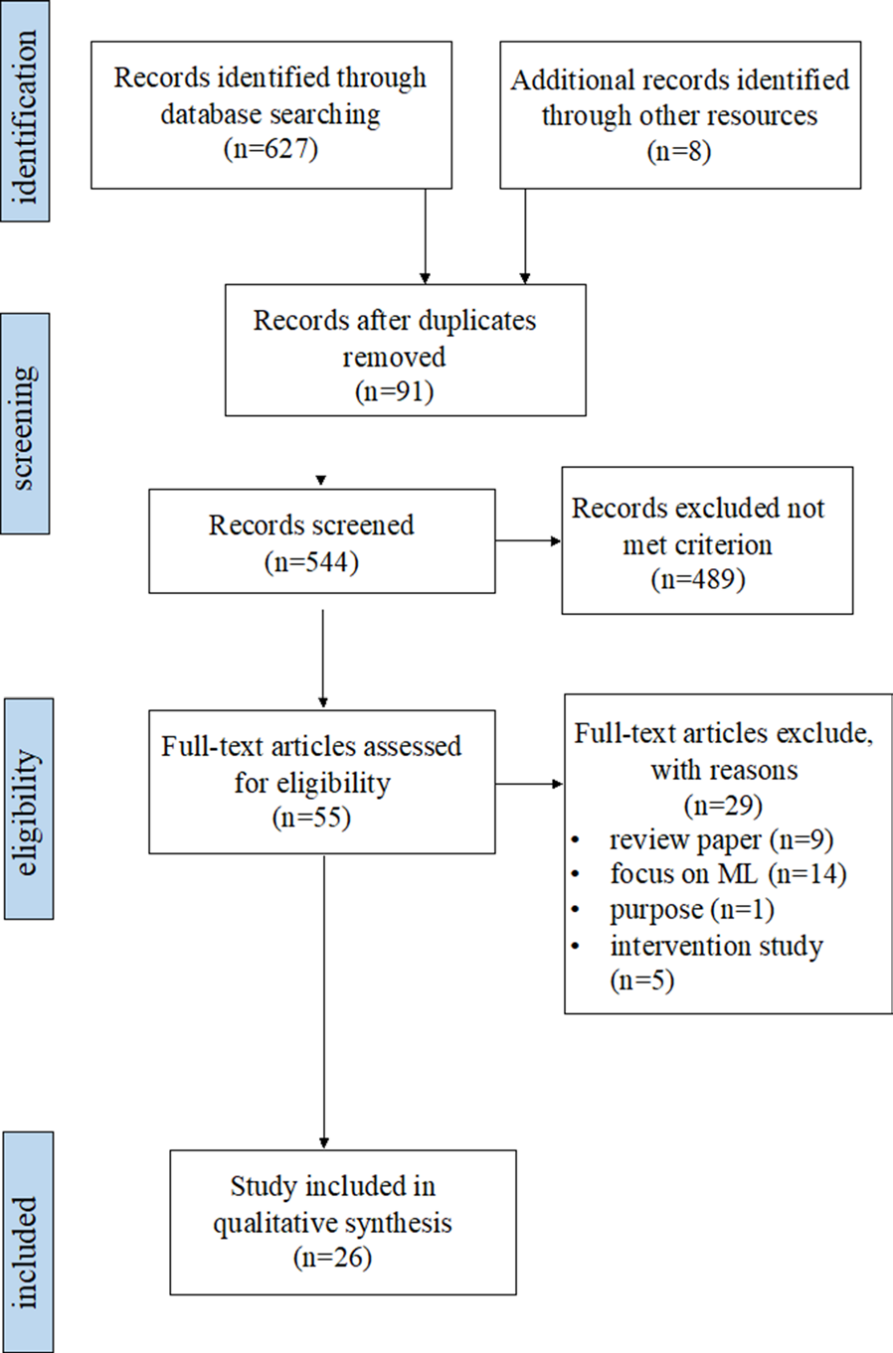
PRISMA flowchart of the study selection process

This study followed the Preferred Reporting Items for Systematic Reviews and Meta-Analyses (PRISMA) guidelines for the systematic review process, and the overall procedure can be divided into four areas: identification, screening, eligibility, and inclusion of articles (see Fig. 1). A systematic search was conducted using the Web of Science, PubMed, ERIC, and PsycINFO databases for articles published from 2000 to June 2022. The keywords telemedicine and autism were used to search the titles and abstracts (“telemedicine” or “telehealth” or “video-based” or “digital device” or “digital health” and “autism”, allowing searches for ASD, autism, ASD, etc.). In addition, searches were limited to articles in English-language, peer-reviewed journals; no restrictions on publication date were applied.

In the four databases, 627 articles containing both telemedicine and autism-related keywords were found; 8 related papers in Google Scholar were also collected. After removing duplicates of 91 articles, a total of 544 articles were obtained. The titles and abstracts of the articles were chosen by the first author based on predetermined inclusion criteria, which included (a) use of telemedicine technology for ASD screening, assessment, or diagnostic purposes; (b) inclusion of quantitative information on the capability of the technology (or the technology-based tool); and (c) publication in English in a peer-reviewed journal from 2000 to June 2022. All types of empirical study designs were included, but studies were excluded if they were (a) not original articles (reviews/meta-analyses, opinion articles, brief reports); (b) did not involve the use of any telemedicine technologies and only presented a discussion of screening or diagnostic ASD tools, and (c) digital health articles regarding ASD interventions or treatments. After screening the 544 articles based on the titles and abstracts, 55 articles were retained. These articles were independently read verbatim by the first and second authors to determine eligibility. Following a consensus-based review, 26 papers met all of the inclusion criteria.

The Scientific Merit Rating Scale was used to conduct a quality review of the included articles (SMRS; National Autism Center, 2015). The review assessed experimental rigor in five main areas: research design, participant ascertainment, measurement of dependent and independent variables, and generalization. An overall scientific merit score was generated. A score of 0–1 indicates insufficient scientific rigor, a score of 2 indicates preliminary evidence indicating positive effects that need to be confirmed using more scientific rigor, and a score of 3, 4, or 5 indicates sufficient scientific rigor for drawing reliable conclusions. Finally, the first author evaluated each study that met the inclusion criteria and extracted the following information from it: author (year), device or technique used, the purpose of the device or technique, tools involved in the device or technique, observed indicators, and applied scenarios.

## Results

### Study characteristics

The search yielded 627 articles; 8 additional articles were obtained from other sources, for a total of 635 articles. After removing 91 duplicated articles, 544 were chosen for title and abstract screening. A total of 55 articles were chosen for full-text review. Following a thorough examination, 29 articles were excluded for the following reasons: treatment or intervention studies only (n = 5); review articles (n = 9); focus on algorithms (n = 14), and studies for purposes other than screening and diagnostics (n = 1). Thus, 26 articles remained in the systematic review and underwent subsequent data extraction (see Fig. 1 and Table 1). The quality of the included articles was assessed using the SMRS, which revealed that the overall quality was good, with a mean score distribution of 3 or more; only two studies received a score of 2, indicating the need for more scientific rigor [40, 45]. Only English-language articles were considered for this study; thus, out of the 26 studies, most were conducted in the US. Two studies were conducted in the United Kingdom, 2 in New Zealand, and 1 in Australia. In addition, of the 17 applications reported in this review, 4 were developed by companies, and 10 were based on university-funded projects. The website for each application is shown in Table 1.

**Table 1:** Summary of study characteristics

	Device	Author (year)		Country	Website	Affiliation	Method	Technology utilized	Diagnosis or screening?	Observed metrics	Assess scenario
1	Cognoa	Kanne et al. [20]		US	https://cognoa.com/	Cognoa Inc	Store-and-forward method	Application	Screening	Caregiver questionnaire (MARA) + health care questionnaire + video coding (behavior features)	3 home videos shoot by their parents, and automatically coding the video
		Abbas et al. [1]		US							
		Abbas et al. [2]		US							
		Megerian et al. [28, 29]		US							
2	Autism & Beyond	Egger et al. [15]		US	https://autismandbeyond.com.au	Duke University	Store-and-forward method	Application	Screening	3 surveys (family background, parental concerns, and temper tantrums) + video coding (face landmark)	Watch short videos and iphones’ camera recording their face, and automatically coding the features
		Campbell et al. [6]		US							
		Carpenter et al. [8]		US							
		Perochon et al. [33]		US							
3	ASDTests	Thabtah et al. [45]		New Zealand	https://www.asdtests.com	Dr. Fadi Fayez in Manukau Institute of Technology	Static scoring	Application	Screening	AQ-Adult-10, AQ-Adolescent-10, AQ-Child-10, and Q-CHAT-10	Parents, care givers, and academic researchers to fill out tests, which are behavioural tests that just pinpoint to autistic traits
4	ASDetect	Barbaro and Dissanayake [5]		Australia	https://asdetect.org	Sadka et al. in Trobe University, Australia	Static scoring	Application	Screening	Questions contain self-developed early markers of ASD behavioral characteristics	Parents watch instruction videos and answer questions
5	AutismAI	Shahamiri and Thabtah [40]		New Zealand	https://www.autismai.com	Seyed Reza Shahamiri and Fadi Thabtah	Machine learning	Application	Screening	AQ and Q-CHAT-10	Users need to answer all the questions and press the submit button and generate a report
6	NA	Wingfield et al. [48]		UK	NA	NA	Machine learning	Application	Screening	The PAAS checklist (pictorial autism assessment schedule)	Users answer questions
7	TEDI	Talbott et al. [44]		US	https://health.ucdavis.edu/mindinstitute/research/telehealth-evaluation-development-for-infants/index.html#:~:text=The%20goal%20of%20the%20TEDI%20study%20is%20to,to%20a%20clinic%20to%20get%20a%20developmental%20evaluation	University of California, Davis	Real-time method	Video conferencing	Screening	AOSI + IGDI-ECI	The TEDI kit includes cue cards and toys to support 10 different interactive “scenarios,” 5 scenarios for specific items from the AOSI, and 5 additional scenarios to stimulate object exploration, play, and communication
8	CHICA	Downs et al. [14]		US	https://medicine.iu.edu/pediatrics/specialties/health-services/child-health-informatics-research-development-lab/the-chica-system	Researchers from the Regenstrief Institute and Indiana University	Static scoring	An automated system	Screening	20 questions + M-CHAT-F	Users answer 20 yes or no questions covering a wide range of primary care issues, 6 questions were selected to alert clinician
9	The video-referenced Infant Rating System for Autism (VIRSA)	Young et al. [49]		UK	NA	University of California, Davis	Machine learning	Application	Screening	Questions contains different level of social communication	The user refers to the videos on both sides of the screen (left and right) and chooses the one that most resembles the child, scoring it on a 10-point scale. The video library includes 268 videos all from 11 children with autism, 23 with non-autism, and 29 with typical development
10	Lena	Oller et al. [32]		US	https://www.lena.org	Infoture Inc	Store-and-forward method	A wearable device	Screening	Assess children’s language development + M-CHAT	The parent or teacher places the LENA device in the child’s LENA undershirt and records the child’s sound environment throughout the day. The device then processes the recordings into data indicators connected to a computer, including the child’s exposure to speech stimuli, the amount of speech the child speaks, and other information
11	SORF	Dow et al. [12]		US	NA	Florida State University	Store-and-forward method	Application	Screening	SORF coding	A videographer is present to record the observation of parent and children, and the parents are given written and verbal instruction to interact with children. The recorded video then coded by trained coders using SORF
12	NODA	Smith et al. [41]		US	https://www.nodaautismdiagnosis.com/	Behavior Imaging Solutions Inc	Store-and-forward method	Application	Diagnosis	DSM-5	NODA consists of 2 components: (1) NODACapture, an app to allow parents to record video evidence of their child’s prescribed behavior at home; and (2) NODA Connect, a web portal that enables physicians to assess the child based on home video and developmental history and by linking evidence of behavior flagged in the video to DSM5 criteria
13	NA	Reese et al. [37]		US	NA	Interdisciplinary Technical Assistance Center on Autism and Developmental Disabilities, University of Kansas	Real-time method	Video conference	Diagnosis	DSM-5 + ADI-R + ADOS-2	Video conference to evaluate ASD
14	NA	Juárez et al. [19]		US	NA	NA	Real-time method	Fixed point-tilt-zoom cameras in clinic rooms/ video conference	diagnosis	STAT + DSM-5 + clinical best estimate (CBE) + MSEL + VABSII + ADOS-2	Use camera or video conference to remote diagnosis
15	The Brief Observation of Symptoms of Autism (BOSA)	[13]		US	https://www.sfari.org/funded-project/the-initial-standardization-of-the-brief-observation-of-symptoms-of-autism-bosa/	Funded by Simons Foundation, Semel Institute for Neuroscience and Human Behavior, University of California, Los Angeles (UCLA)	Real-time method	Video conferencing	Diagnosis	Four versions of BOSA (PSYF, MV, F1, F2)	A 12–14 min interaction between an individual and a caregiver or clinician, materials are picked based on age and language and developmental level, and a trained clinician observe and code base on the BOSA manual
16	TeleNP	Ransom et al. [36]		US	https://www.teladochealth.com	Teladoc Health Inc	Real-time method	A video-based practice model	Screening and diagnosis	DSM-5 + CARS-2 + NEPSY-II + DKEFS + Vineland-3 + ADOS-2	Verbal instructions were provided to families by telephone to set environment, 20- to 30-min child interview and observation online, different assessments conducted via teleNP
		Salinas et al. [39]		US							
17	The TELE-ASD-PEDS	Stavropoulos et al. [42, 43]		US	https://triad.vkclearning.org/	Vanderbilt Kennedy Center: Treatment and Research Institute for Autism Spectrum Disorders (TRIAD)	Real-time method	Video conferencing	Assessment	ADOS-2 (Modules 2 and 3)	Module 2 (15 Activities and 9 codes), module 3 (11 Activities and 10 codes)
		Corona et al. [10]		US							
		Wagner et al. [47]		US							

*AQ* The Autism-Spectrum Quotient Test, *Q-CHAT* The Quantitative Checklist for Autism in Toddlers, *PAAS Checklist* Pictorial Autism Assessment Schedule checklist, *AOSI* The Autism Observation Scale for Infants, *IGDI* Individual Growth and Development Indicators, *ECI* Early Childhood Intervention, *M-CHAT* The Modified Checklist for Autism in Toddlers, *ADI-R* The Autism Diagnostic Interview-Revised, *ADOS-2* The Autism Diagnostic Observation Schedule, Second Edition, *STAT* Screening Tool for Autism in Toddlers & Young Children, *CBE* clinical best estimate, *MSEL* Mullen Scales of Early Learning, *VABSII* Vineland Adaptive Behavior Scale, *CARS-2* Childhood Autism Rating Scale, Second Edition, *NEPSY-II* NEPSY-Second Edition, *DKEFS* Delis–Kaplan Executive Function System, *Vineland-3* The Vineland Adaptive Behaviour Scales, Third Edition, *ESAC* the Early Screening for Autism and Communication Disorders, *MSEL* Mullen Scales of Early Learning, *VABS-2* Vineland Adaptive Behavior Scale-Second Edition

### Telehealth method

Telemedicine provides access to expert medical care and support without the need to meet in person [4]. When using a video-based approach to screen for ASD, two main technological approaches apply: real-time and store-and-forward methods. Real-time interactions allow patients to communicate with healthcare providers in real time, primarily through video conferencing, whereas store-and-forward interactions do not necessitate the simultaneous presence of patients and healthcare providers [27]. Scales and questionnaires used to remotely screen for ASD can be divided into two approaches: static scoring and machine learning (ML). Static scoring is the online use of conventional questionnaire scoring, while machine learning is the use of online ML algorithms to score questionnaires more individually and flexibly [40]. This review elaborates on telemedicine studies in ASD focusing on three purposes: screening, assessment, and diagnosis. Some applications can satisfy more than one purpose.

### Screening

Eleven ASD screening applications were summarized in this study. Overall, most of the applications are well-established. Cognoa, Autism&Beyond, ASDtests, ASDetect, AutismAI, and VIRSA are all mobile-based applications that can be downloaded directly to a smartphone or tablet [5, 15, 20, 40, 45, 49]. Although the TEDI (Telehealth Evaluation of Development for Infants), CHICA (Child Health Improvement through Computer Automation), Lena and SORF (the Systematic Observation of Red Flags) applications are not web-based, empirical studies have demonstrated the feasibility and validity of these tools [7, 13, 14, 44]. TEDI is a novel tool for remotely assessing the behavioral characteristics of children with ASD, and CHICA is a computer-automated decision support system that combines screening questions and medical history data displayed on a tablet to conduct remote screening for ASD. In addition, one more application was recommended but has not been adequately developed [48]. These tools involved four different types of processing, namely, (a) static scoring, (b) machine learning, (c) storage and uploading, and (d) real-time methods. Table 1 illustrates the methods used for each screening application.

Four applications used the store-and-forward approach (Cognoa, Autism&Beyond, Lena, and SORF), and one application used the real-time approach (TEDI). Cognoa is a machine learning-based application that analyzes two short (1 min and 30 s to 5 min) home videos recorded by parents, along with caregiver questionnaires and clinical question sets from primary care physicians, to generate “ASD positive,” “ASD negative,” or “no result” reports [28, 29]. The Autism&Beyond application mainly uses the camera to record the child’s facial features while watching a short video and combines surveys to screen for ASD [6]. Lena is a wearable device that allows teachers or parents to record a child’s language environment remotely and analyzes recorded language information features to screen for ASD [32]. SORF is an observational coding system for identifying red flags (RFs) for ASD in young children, also allowing remote screening for ASD via naturalistic video-recorded home observation [13]. Parents receive verbal and written instructions for interacting with their children and engage in suggested activities for approximately 5–10 min per day for no more than 1 h. Behaviors in the videos are coded by trained coders using SORF to predict ASD risk. Only one tool, TEDI, employed the real-time approach. TEDI uses video conferencing to screen ASD by directly observing children’s interactions during 10 activities [44].

The other six screening tools used scales or questionnaires to screen for ASD, with ASDTests, ASDetect, and CHICA scoring questionnaires statically only and AutismAI, VIRSA, and Wingfield’s application all using machine learning to score [5, 14, 40, 45, 48, 49]. Compared to static scoring, the machine learning approaches yielded slightly higher sensitivity and specificity. For example, AutismAI reported a sensitivity of 0.955 and a specificity of 0.986, and ASDTests reported a sensitivity ranging from 0.922 to 0.98 and a specificity ranging from 0.85 to 0.997. The machine learning application proposed by Wingfield et al. [48] used the PAAS checklist (Picture Autism Assessment Scale) to screen ASD in infants aged 2–4 months and reported a sensitivity of 0.88 and a specificity of 0.96. VIRSA, on the other hand, uses videos instead of questions to allow caregivers to select the most relevant videos of the child’s behavior; thus, is easier for the user to understand and make more accurate judgments. VIRSA was reported to be more sensitive for ASD screening (sensitivity = 1).

### Diagnosis

One application and three other studies refer to remote ASD diagnosis. NODA is a well-established application that can be downloaded and used directly. Other studies only investigated the feasibility of telemedicine applications from different perspectives. Among them, the BOSA (The Brief Observation of Symptoms of Autism) approach proposed in the study by Dow et al. [13] is very promising, but further research is still needed. NODA (Naturalistic Observation Diagnostic Assessment) is a store-and-forward application that consists of 2 main components [41]. (1) NODA Capture enables parents to use their mobile phones to upload short videos of their concerns and their child’s behavior in specific situations, such as family meals, playtime with others and playtime alone, for sharing remotely with a clinician. The child’s play-based behaviors and social communication abilities are primarily shown in the first three scenarios, while the parent’s concern about the child’s behavioral symptoms is primarily shown in the final scenario. Parents set up the environment according to the provided scenario descriptions and sample videos. All recordings (including the child’s developmental history) are uploaded to the portal. (2) NODA Connect is a portal that enables physicians to diagnose the child based on the home video and developmental history and to link the marked behavioral characteristics in the video (e.g., no eye contact) with DSM-5 criteria. It is possible to inform parents and other medical professionals about the diagnosis findings through this portal [41].

For ASD diagnosis, Reese’s, Juárez’s and Dow’s teams all used real-time video conferencing [19, 37]. The Reese et al. study was assessed primarily by combining (a) 10 min of observation of the child’s performance in an unstructured setting, (b) activities from the revised ADOS-2, (c) a structured interview using the ADI-R (Autism Diagnostic Interview-Revised), (d) a medical history review, (e) feedback and advice to the family, and (f) a satisfaction survey. Juarez et al. assessed ASD using (a) a brief interview, (b) the implementation of STAT (Screening Tool for Autism in Toddlers & Young Children), and (c) a focused diagnostic ASD interview. Clinicians interviewed caregivers directly via video conferencing and observed the child’s behavior at STAT for ASD diagnosis. The diagnostic procedure of the Dow et al. study included (a) the ADOS–Toddler Module (ADOS-T), (b) a 1-h home observation coded by the BOSA (The Brief Observation of Symptoms of Autism) manual, (c) assessment of developmental level using the Early Screening for Autism and Communication Disorders (ESAC) and Mullen Scales of Early Learning (MSEL), and (d) adaptive behavior assessed by the Vineland Adaptive Behavior Scale-Second Edition (VABS-2) from a caregiver interview.

The main difference between the two studies was that Reese et al.’s study focused on comparing real-time video conferencing with an in-person assessment to show the feasibility and reliability of the online ASD assessment, while Juárez’s study provided a detailed description of the procedure for telemedicine diagnosis of ASD. Reese et al.’s study found that there was still excellent diagnostic agreement between clinicians and other teams in the video conferencing setting. Real-time videoconferencing achieved the same results (sensitivity = 0.84, accuracy = 0.88) as the psychometric properties of in-person assessments (sensitivity = 0.88, accuracy = 0.78), consistent with a previous review [37, 42, 43]. In contrast, Juárez et al. studied the preliminary feasibility, accuracy, and clinical utility of diagnosing ASD via televideo conferencing. This study showed that telemedicine procedures were as capable as in-person assessments at identifying children diagnosed with ASD. Although 13% of the participants could not provide a diagnosis [19], it is undeniable that video conferencing could improve the efficiency of ASD diagnostics.

Another application that also uses real-time video conferencing is BOSA, which was proposed by Dow et al. [13]. BOSA begins with a 12–14-min interaction between the individual and a clinician or caregiver, followed by observation by the trained clinician via videoconference and objective coding according to the BOSA manual, leading to a diagnosis of ASD. This application has the advantages of ADOS-2 in that it allows a relatively natural and consistent context to observe social-communicative behaviors and perform standardized coding while avoiding the drawbacks of ADOS-2, such as the long diagnostic time and the inability to perform remote assessments [13].

### Screening and diagnosis

One application, TeleNP, performs both screening and diagnostic functions. TeleNP is a real-time practice model encompassing almost the entire patient assessment process from the initial screening visit to the subsequent diagnostic visit and is currently being evaluated in two studies [36, 39]. This complete model includes the process of online clinic intake, history collection, appointment visits, and diagnostic assessment. First, patients can upload information directly to the online platform to generate an electronic medical record. After receiving the medical file, a questionnaire for an ASD screening appropriate for the child’s age and area of concern is sent via email. Caregivers then complete the questionnaire and are given verbal instructions over the phone regarding how to create a distraction-free environment to maximize the child’s participation in the testing procedure. The ideal room or seating area is determined based on the child’s behavior. For example, sitting on the parent’s lap in the living room or setting up the bedroom with preferred toys on the floor. The examiner asks the parents about the child’s preferred toys and any fixations during the appointment to help promote a positive relationship and reduce the likelihood of emotional outbursts. Finally, after a 20-to 30-min child interview and observation period, the examiner conducts a 20-to 30-min rapid diagnostic interview with the caregiver based on DSM-5 criteria.

### Assessment

Only one instrument, which is neither a screening nor a diagnostic tool, is used to assess ASD core behaviors. TELE-ASD-PEDS is a core behavioral remote assessment tool developed for young children at high risk for ASD and has been discussed in three studies [10]. TELE-ASD-PEDS assesses ASD via real-time video conferencing and focuses on young children aged 1–3 years who are not yet verbal. Trained examiners assess children based on their behaviors during different activities via real-time video conferencing. A machine learning algorithm selects the 12 most predictive ADOS-2 activities and applies them to a parent-led interactive task. During implementation, clinicians rate the child’s behavior on seven items via video conferencing (e.g., eye contact and unusual repetitive restricted interest/stereotyped play) and use a dichotomous (yes/no) and a Likert scale to rate the severity of symptoms (3 = behavioral features of ASD present; 2 = probably atypical behavior; 1 = behavioral features of ASD not present). Although the TELE-ASD-PEDS is not designed for screening and diagnosing ASD, preliminary data suggest that it is a useful and valid ASD diagnostic tool [42, 43].

### Observed behavioral indicators

ASD screening, diagnosis, or assessment applications rely on ASD behavioral indicators to allow parents to report a symptom or for a clinician to give a score. Although the 17 applications had different purposes, the behavioral indicators they observed fell into two broad categories: comprehensive and noncomprehensive. Comprehensive behavioral indicators usually cover the full dimension of ASD characteristics stated in DSM-5, including social communication and interaction deficits, restricted and repetitive patterns in behavior and interest, developmental histories and related information. Noncomprehensive observed indicators only focus on some core behavioral characteristics of ASD.

Most applications reviewed here used comprehensive observational indicators, and three studies used noncomprehensive observational indicators in the screening tools. It is worth noting that most of the screening tools employed extensive observational indicators. Cognoa, a screening tool, not only collected information from the caregiver’s perspective and flagged core behavioral characteristics from videos but also received additional information from clinicians for screening. Moreover, TEDI used a real-time approach to observe different core behavioral characteristics in 10 scenarios, and SORF used the store-and-forward method to collect videos to code 22 items, with 11 items in each of the two DSM-5 symptom domains—Social Communication (SC) and Restricted Repetitive Behaviors (RRB). ASDTests, ASDetect, AutismAI, CHICA, and the studies by Wingfield et al. used established screening tools or combined them with their developed questionnaires, with the most commonly used screening tools being The Modified Checklist for Autism in Toddlers (M-CHAT) and the 10-item Autism-Spectrum Quotient (AQ-10).

All diagnostic tools used a comprehensive set of observables. The DSM-5 diagnostic criteria were used by NODA, TeleNP, and the tool proposed by Juárez et al. to diagnose ASD. Although BOSA uses a standardized coding manual developed in-house, the score still corresponds to the DSM-5 checklist and the ADOS-2 score. Moreover, TeleNP also used the childhood autism rating scale (CARS-2), the NEPSY second edition (NEPSY-II), Delis-Kaplan executive function system (DKEFS), vineland adaptive behavior scales, third edition (VABS-3), and autism diagnostic observation schedule, second edition (ADOS-2). Juárez et al. used STAT, clinical best estimate (CBE), Mullen scales of early learning (MSEL), VABS-2, and ADOS-2 as other observables. The most commonly used diagnostic tool is the ADOS-2. Assessment tools such as the TELE-ASD-PEDS and the study by Reese et al. also used a full range of observables, both of which used the ADOS-2; Reese et al. also used the Autism Diagnostic Interview-Revised (ADI-R) for assessments.

Only three screening applications used noncomprehensive observables. Autism&Beyond, Lena, and The Video-referenced Infant Rating System for Autism (VIRSA) examine only one core behavioral characteristic of ASD [8, 32, 49]. Autism&Beyond examines the child’s facial features and uses an algorithm to code the child’s social emotions while watching the video. Lena focuses on child’s language environment and screens ASD from a language development perspective. VIRSA, on the other hand, assesses ASD from the standpoint of social skills. A total of 268 videos of social activity were collected so that caregivers could view them in a mobile app and choose the one that best matched the child’s social communication behavior.

### Psychometric properties

We identified 26 studies describing the telemedicine application they used and their observed indicators for screening, assessing, or diagnosing ASD. Table 2 shows the demographic information (e.g., sample size, age and sex) of the individuals studied and key information regarding the instruments’ psychometric properties. The age range of the participants in the studies varied widely. Fourteen studies included children between the ages of 18 and 72 months, 4 included children between the ages of 3 and 18 months, 6 included children with ASD aged 6 or older, and 2 studies did not provide sufficient information. Seventeen studies had > 50% male representation, and sex information was missing in 4 studies (see Table 2). Four studies reported all four metrics considered relevant to the instrument (i.e., sensitivity, specificity, positive predictive values and negative predictive values), and 15 papers provided data on at least one psychometric property. Among the studies reporting psychometric information (n = 15), 8 reported sensitivity and specificity values equal to or greater than 75%. However, it should be noted that sensitivity values below this threshold may not indicate poor psychometric properties as the tool may be reliable for detecting specific subgroups of ASD patients [11, 34].

**Table 2:** Analysis of the studies included in the review

	Device	Author (year)	Sample	Age	Gender	Psychometric properties			
						Sensitivity	Specificity	PPV	NPV
1	Cognoa	Kanne et al. [20]	164 ASD + 66 non-ASD	18–72 months	81% ASD male + 75.7% ASD male	0.75	0.62	0.83	0.5
		Abbas et al. [1]	162	18–72 months	NA	0.982	0.624	NA	NA
		Abbas et al. [2]	375	18–72 months	NA	0.9	0.69	NA	NA
		Megerian et al. [28, 29]	425	18–72 months	64% male	0.98	0.79	0.81	0.98
2	Autism & Beyond	Egger et al. [15]	1756	Mean age at 40.4 month	23.2% male	NA	NA	NA	NA
		Campbell et al. [6]	22 ASD + 82 TD	16–31 months	NA	0.96	0.38	NA	NA
		Carpenter et al. [8]	22 ASD + 74 TD + 8 non-ASD	16–31 months	62.5% male	NA	NA	NA	NA
		Perochon et al. [33]	856TD + 37ASD + 17LD-DD	17–37 months	49.34% male	NA	NA	NA	NA
3	ASDTests	Thabtah et al. [45]	20	NA	70% male	0.922–0.98	0.85–0.997	NA	NA
4	ASDetect	Barbaro and Dissanayake [5]	39 AD + 50 ASD + 20 DD	12, 18, 24 months	NA	NA	NA	NA	NA
5	AutismAI	Shahamiri and Thabtah [40]	6075	NA	58% male	0.955	0.986	NA	NA
6	NA	Wingfield et al. [48]	86 control + 195 ASD	2–4 months	82.56% male	0.88	0.96	NA	NA
7	TEDI	Talbott et al. [44]	11	3–11 months	45.45% male	NA	NA	NA	NA
8	CHICA	Downs et al. [14]	274	23–30 months	59.12% male	NA	NA	NA	NA
9	The Video-referenced Infant Rating System for Autism (VIRSA)	Young et al. [49]	73 ASD history + 37 non-ASD history	6, 9, 12, 18 months	Around 61.91% male	1	0.53	1	0.19
10	Lena	Oller et al. [32]	106 TD + 49 LD + 77 ASD	10–48 months	Around 74% male	0.75	0.98	NA	NA
11	SORF	Dow et al. [12]	84 ASD + 144 TD/DD	18–22 month	72.8% male	0.70	0.67	0.55	0.79
12	NODA	Smith et al. [41]	11 TD + 40 ASD	18 month-6 years 11 months	70.59% male	0.85	0.94	NA	NA
13	NA	Reese et al. [37]	17	2.5–6 years	29.4% male	0.84	0.88	NA	NA
14	NA	Juárez et al. [19]	study 1 = 20, study 2 = 45	20–34 months	80% male, 77.78% male	0.79	NA	NA	NA
15	BOSA	[13]	307	15 months–42 years	74% male	0.86–0.96	0.70–1	NA	NA
16	TeleNP	Ransom et al. [36]	129	1–21 years	48.84% male	NA	NA	NA	NA
		Salinas et al. [39]	67	2–18 years	55.22% male	NA	NA	NA	NA
17	The TELE-ASD-PEDS	Stavropoulos et al. [42, 43]	23	81.7 month	78.3% male	NA	NA	NA	NA
		Corona et al. [10]	35 ASD + 10 DD + 6 TD	2.51 month	70.59% male	NA	NA	NA	NA
		Wagner et al. [47]	204	27.54 month	77% male	NA	NA	NA	NA

Eight screening tools and four diagnostic tools existed among the 15 studies that reported psychometric attributes. Of the eight studies with screening tools, only the ASDTests study had a small sample size (n = 20), while the others were above 100. VIRSA had the highest sensitivity (sensitivity = 1), AutismAI, ASDTests, Autism&Beyond, and Cognoa had great sensitivity (exceeding 0.9), Wingfield’s proposed application’s sensitivity was 0.88, and Lena’s was 0.75. SORF had the lowest sensitivity (0.70). The proposed applications from AutismAI, ASDTests Lena, and Wingfield all had good specificity (> 0.9); Cognoa, SORF, VIRSA, and Autism&Beyond had specificities of 0.79, 0.67, 0.53, and 0.38, respectively.

Of the four studies of diagnostic tools, only the study by Dow et al. [12] had a sample size greater than 100, while the other three studies had sample sizes less than 100, with Reese et al. [37] including only 17 participants. In addition, the study by Dow et al. had a better sensitivity (0.86–0.96) than that by Reese et al. [37] (0.88), while the application studied by NODA and Juárez et al. [19] presented a sensitivity between 0.79 and 0.85. However, both NODA and Juárez et al. [19] reported specificities greater than 0.94, while the specificity of BOSA fluctuated between 0.70 and 1.

## Discussion

This paper focused on the accumulating evidence regarding ASD screening, diagnostic and assessment applications, covering the technology utilized, observed metrics, and assessment scenarios of those applications and the sample characteristics and psychometric properties described in the studies. We included 26 studies covering 17 telehealth applications. We first summarized the methods used by the 17 applications, revealing that screening, diagnostic, and assessment tools could be classified as real-time or store-and-forward, static scoring or machine learning based on content-based (video-based or scale-based) approaches. Then, we compiled the behavioral observation indicators and discovered that the majority of applications made use of thorough observation indicators, i.e., screening, assessment, or diagnosis, using the most recent diagnostic criteria and combined with well-established scales or questionnaires. A few applications used noncomprehensive observables, assessing only one core behavioral characteristic of ASD. Finally, we summarized the demographic information and psychometric properties of the 26 studies, and the results indicated that approximately half of the studies did not report their psychometric properties. The benefits, drawbacks, and potential future developments of telehealth applications are outlined in the sections below.

### Strengths of telemedicine applications

As stated in the introduction, telemedicine has numerous advantages, which were well demonstrated in the studies included. First, telemedicine can help address the growing need for the rapid screening, assessment, and diagnosis of ASD, which is one of the goals of many telemedicine applications. This study reviewed 11 screening tools, 4 diagnostic tools, 1 tool that can be used for both screening and diagnosis, and 1 assessment tool. Of these 17 tools, 60% were apps with websites that can be employed directly for appointments or downloaded to a cell phone, which can cut wait times down to a few weeks or days from up to 18 months. Although some tools, such as TELE-ASD-PEDS, require parent-led interaction, studies have shown that parents and clinicians are sufficiently comfortable using this type of remote assessment [10].

Second, telemedicine applications can greatly improve the standardization of ASD screening, assessment, and diagnosis in primary care settings with good sensitivity and specificity. Currently, only approximately 1% of ASD patients in the United States are diagnosed by primary healthcare providers, and telemedicine applications with portable and easily accessible devices can greatly improve this situation [30, 38]. For example, Cognoa employed the store-and-forward approach to collect caregiver-uploaded videos and caregiver and clinician questionnaires and combined the machine learning algorithm to determine ASD risk, achieving a sensitivity of 0.98 and specificity of 0.79 [20]. Similarly, NODA, which used caregiver-uploaded videos for clinicians to diagnose ASD, had a sensitivity of 0.85 and a specificity of 0.94 [41]. In contrast, using a real-time approach, video conferencing allows clinicians to screen or diagnose by directly observing the behavioral characteristics of children as they interact with their parents. One study by Reese et al. [37] found that remote diagnosis also had good psychometric properties compared to face-to-face visits, with a sensitivity of 0.84 and a specificity of 0.88.

Third, digital health approaches save time and are becoming more efficient than other existing tools, such as ADOS, which can take up to 45 min to complete, not including the travel time to the medical facility. Digital health applications such as BOSA maintain the benefits of ADOS but reduce the time required to 10–12 min [13]. Cognoa, on the other hand, only takes 10 min to register information, fill out a questionnaire with a few questions, and upload two or three videos before obtaining a complete ASD screening report [2]. Autism&Beyond is even more convenient, requiring children to watch a total of approximately 2 min of short videos for facial data collection and only approximately 20 min of questionnaire completion for caregivers. Therefore, a significant amount of time is saved [33]. In addition, digital health can help address parents’ concerns about contracting COVID-19 during travel to and from clinics given the pandemic circumstances. While preventing COVID-19 infection may not be the purpose of most telemedicine applications, it can still have a positive preventive effect. Whether an app or video conferencing is used, digital health technology allows users and clinicians to screen, evaluate, or diagnose during an epidemic, and remote diagnosis eliminates the need for face-to-face communication.

### Recommendations for telehealth

There are many things to consider when choosing an ASD telehealth application, such as the aforementioned time required, comprehensive observation indicators, and ease of access to the application. Overall, both Cognoa and AutismAI are optimal choices for early screening alone. Both applications use comprehensive observational indicators, and the assessments are short and easy to access; recent studies have shown that both have a sensitivity and specificity of ~ 0.8 or higher [28, 29, 40]. For diagnostic purposes, NODA is by far the best choice, again using a comprehensive set of observables that are easy to obtain, and studies have shown good measurement properties (sensitivity of 0.85 and specificity of 0.94) [41]. Notably, the BOSA platform holds promise, but further empirical validation will be required once it progresses beyond its current pre-development stage [13].

### Limitations and considerations

Despite the numerous benefits of the previously mentioned telemedicine applications, the studies that validated them have some serious limitations. A major issue in studies evaluating ASD screening and diagnostic tools is that not all studies reported on their psychometric properties. Of the 26 included studies, 15 (58%) reported at least one psychometric attribute, while the remaining 11 (42%) did not. Those studies that did not report mainly focused on other aspects. For example, Talbott (2019) et al. examined the reliability, validity, and feasibility of the TEDI. Barbaro and Dissanayake [5] mainly examined the core behavioral characteristics of children with ASD at 12 months, 18 months, and 24 months as the primary observational indicator. In addition, three studies discussed the accuracy of Autism&Beyond screening for ASD or conducted ROC analyses. However, as previously mentioned, it is impossible to assess the validity of the tool in this situation. Some studies simply examined user acceptability and satisfaction with the tool. Ransom et al. [36] used ANOVA to examine user acceptance of the TeleNP tool in different media (mobile/computer), and Downs et al. [14] used CHICA as a tool to assess the effectiveness of the intervention approach.

While AutismAI covered all age groups (young children, adolescents, and adults) for screening ASD, 2 studies out of the 15 that reported psychometric properties did not report any information about the participants’ ages, potentially due to the group that the application targets. Four studies did not report information on the sex of the participants. The studies by Shahamiri and Thabtah did not include a non-ASD group as part of the study, hence only reporting the sensitivity value. In addition, positive predictive values (PPV) and negative predictive values (NPV) were reported in only four studies. Therefore, future studies should include complete reporting of instrument psychometric properties to make the studies more rigorous.

The poor performance of the psychometric properties reported by these applications is another issue with these telehealth studies. The psychometric properties of the screening tools showed an overall high sensitivity and low specificity. For example, the latest study of Cognoa showed a sensitivity of 0.98 and a specificity of 0.79 for ASD screening in 425 children aged 18–72 months [28, 29]. The same pattern was observed with Autism&Beyond, which had an ASD screening sensitivity of 0.96 [33]. One possible reason for this finding could be interference and a lack of standardization of the observations. For example, the lack of standardization in the video content and the inconsistent/variable quality may be one of the causes of the low specificity in Cognoa’s analysis of videos uploaded by parents. Autism&Beyond analyzes the child’s facial expressions when watching a short video; therefore, whether the child’s response is caused by the video stimulus or by other distractions may also affect the specificity. The elevated specificity of diagnostic tools such as NODA (0.94) made the standard administration of the tools more convincing, as the NODA observations are somewhat standardized and require the parent to record behaviors predetermined by the examiner in addition to behaviors the parent is concerned about [41].

## Future direction

Although numerous telehealth programs for various ASD applications have been created, empirical studies of these applications are still relatively insufficient. The current review only included English-language articles, indicating a potential regional imbalance because most studies were produced in Western nations (the USA, UK, New Zealand, and Australia). Therefore, future studies should consciously cover a wider geographical area, such as Europe and Asia, and examine the applicability of these applications in different countries, which will aid in the advertisement and acceptance of the applications.

The review of these ASD screening tools revealed that telemedicine applications of ASD screening were becoming increasingly briefer, more easily understood by caregivers, and more accessible. Future telehealth applications for screening should also maintain these merits. In addition, machine learning approaches contribute to more individualized assessments and a more accurate report, which could be considered in future applications. It should also be noted that, in addition to providing results, the screening tool should also provide options for the next steps, such as options for further referral or options to make an appointment with providers.

Future telemedicine applications for ASD diagnosis could be improved by including comprehensive observations, preferably using the latest diagnostic criteria combined with parent-administered questionnaires and direct observation of the child. Most importantly, the diagnostic application requires the participation of a physician and cannot be used in isolation. In addition, more rigor is needed in empirical studies of ASD diagnostic tools. For example, a larger sample size and complete demographic information of the whole sample is recommended. If the instruments need to be validated, their psychometric properties should also be reported.

## Data Availability

Not applicable.
