# Correction: A systematic review of telehealth screening, assessment, and diagnosis of autism spectrum disorder

**DOI:** 10.1186/s13034-022-00520-8

**Published:** 2022-11-17

**Authors:** Meimei Liu, Zenghui Ma

**Affiliations:** 1 Vrije University Amsterdam, Amsterdam, The Netherlands; 2 Beijing ALSOABA Technology Co. LTD, ALSOLIFE, Beijing, China

## Correction: Child and Adolescent Psychiatry and Mental Health (2022) 16:79 https://doi.org/10.1186/s13034-022-00514-6

Following publication of the original article [1], the authors identified the errors in the author group. The given and the family names of the authors have been swapped in the original publication of the article.

The incorrect author names: Liu Meimei and Ma Zenghui.

The correct author names: Meimei Liu and Zenghui Ma.

The author group has been updated above and the original article [1] has been corrected.
